# Editorial: Role of entomopathogenic fungi in sustainable agriculture

**DOI:** 10.3389/fmicb.2024.1504175

**Published:** 2024-12-17

**Authors:** Perumal Vivekanandhan, Lucy Alford, Patcharin Krutmuang

**Affiliations:** ^1^Office of Research Administration, Chiang Mai University, Chiang Mai, Thailand; ^2^Department of Entomology and Plant Pathology, Faculty of Agriculture, Chiang Mai University, Chiang Mai, Thailand; ^3^School of Biological Sciences, University of Bristol, Bristol, United Kingdom

**Keywords:** entomopathogens fungi, microbial insecticide, entomopathogenic fungi mode of action, *Tuta absoluta* (Meyrick), ecofriendly insecticide, *Beauveria bassiana* (Ascomycota: Hypocreales), *Metarhizium anisopliae*

In the quest for sustainable agricultural practices, entomopathogenic fungi (EPF) have emerged as highly promising agents for both pest management and soil health enhancement (Skinner et al., 2014). These EPF operate by parasitizing and killing insect pests, effectively reducing their populations without the need for harmful chemical pesticides [Han et al.; Smagghe et al., 2023; Vivekanandhan et al.(a); Shahbaz et al., 2024]. By offering a natural and environmentally friendly alternative, EPF help minimize chemical use and the associated negative impacts on ecosystems. Their role extends beyond pest control, with EPF also contributing to soil health by promoting microbial diversity and suppressing harmful pathogens. This dual benefit supports global efforts to reduce agrochemical dependence, restore and enhance ecological balance, and advance sustainable agricultural practices (Kumar et al., 2024; Zhou et al.).

## Entomopathogenic fungi: a sustainable solution

**Figure 1:**
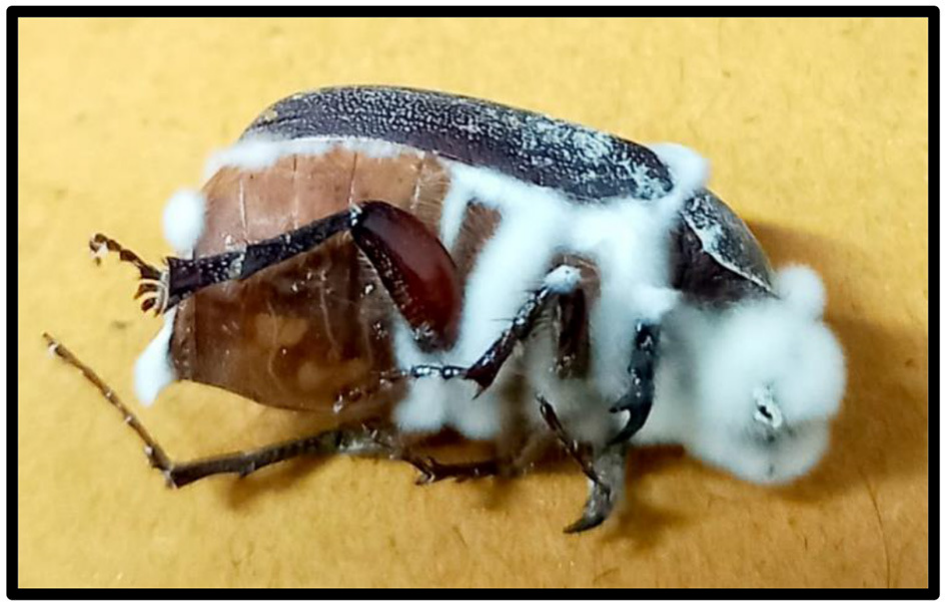
Natural infection of *Beauveria bassiana* on insect hosts. This image captures the fungal pathogen *B. bassiana* infecting its host, showcasing typical mycelial growth and sporulation on the insect body. The infection process involves fungal spores adhering to the host cuticle, penetrating through the exoskeleton, and proliferating within the insect hemocoel. Eventually, the fungus emerges externally, forming white conidial layers on the insect surface, a hallmark of *B. bassiana* infection. This characteristic growth pattern highlights the effectiveness of *B. bassiana* as a biological control agent in insect pest management.

Entomopathogenic fungi, including *Beauveria bassiana, Metarhizium anisopliae, Isaria fumosorosea, Cordyceps* spp, *Entomophthora muscae, Entomophaga grylli*, and *Nomuraea rileyi*, serve as natural enemies of insect pests. These EPF initiate infection by penetrating the insect's cuticle with their spores, before proliferating within the host's internal tissues (Figure 1). Once inside, the fungi are capable of disrupting physiological functions, and producing enzymes and toxins that degrade tissues and suppress the immune system, eventually resulting in insect death [Vivekanandhan et al.(b)]. Furthermore, some EPF are capable of releasing metabolites that alter insect behavior in such a way as to enhance pathogenicity. In doing so, EPF offer a targeted, eco-friendly pest control method that sustainably reduces pest populations [Vivekanandhan et al.(b)].

The use of EPF as a biological control mechanism is highly effective at reducing pest populations while having minimal impact on non-target organisms, including beneficial insects [Vivekanandhan et al.(a); Reddy et al., 2024]. With recent research leading to the discovery of new species, for example of the *Diversispora* and *Scutellospora* genera (Niezgoda et al.), as well as exploring their application to the control of novel insect pests such as the Phasmatodea (Min et al.) and Drosophilid flies [Vivekanandhan et al.(b)], EPFs represent a rapidly advancing field of pest control and a viable alternative to chemical pesticides capable of promoting more sustainable agricultural practices, enhanced soil health, and ecological balance.

## Challenges and future directions

Despite their potential, several challenges hinder the widespread adoption of EPF in agriculture. One major challenge is the variability in efficacy caused by environmental factors such as humidity, temperature, and soil conditions, which can affect the fungi's ability to infect and kill pests (Liu et al.; Min et al.). Furthermore, new research is expanding current understanding of the role that symbiotic bacteria may play in the differential susceptibility of insects to EPF infections and thus EPF efficacy in insect pest control. There is also a need for extensive field trials to understand how these fungi interact with pest species under diverse conditions (Min et al.), as well as interactions with different crops and the potential for mycotoxin contamination, which can be both time- and resource-intensive. Future research must address these limitations by developing more robust and cost-effective fungal formulations that can withstand various environmental conditions and improve their overall performance. The isolation of crude chemical extracts from EPF, enhancing our understanding of the active chemical molecules responsible for insecticidal properties e.g. the larvicidal effect of 9,10-octadecadienoic acid against *Tuta absoluta* (Vivekanandhan, Swathy, Alahmad et al.), may take us closer to this end. We extend our sincere thanks to all authors for their valuable contributions and participation. Additionally, we are deeply grateful to the reviewers and editors, whose dedication and expertise during the publication process were instrumental in the development of this Research Topic.
